# Force-Extension Curve of a Polymer Chain Entangled with a Static Ring-Shaped Obstacle

**DOI:** 10.3390/polym14214613

**Published:** 2022-10-30

**Authors:** Qihao Zhang, Jianfeng Li

**Affiliations:** The State Key Laboratory of Molecular Engineering of Polymers, Department of Macromolecular Science, Fudan University, Shanghai 200433, China

**Keywords:** polymer entanglement, entropic force, topological constraint

## Abstract

The way to theoretically approach dynamic and static topological constraints of polymer entanglements still presents a great challenge in polymer physics. So far, only the problem of static entanglement with multiple simple objects has been solved in theory by a superspace approach in our previous work. This work is devoted to extending the superspace approach to study a polymer chain entangled with a relatively complicated object—a ring-shaped object with genus one. Taking advantage of the axial symmetry of the model setup, the 3D diffusion equations in the superspace can be numerically solved within the 2D coordinates using a specially designed alternating-direction implicit (ADI) scheme. A series of numerical calculations reveal that the topological entanglement effect of the ring will exert a topological entropy attractive force on the linear chain, which can be used to explain the viscosity-increase phenomenon observed in recent simulations and experiments. Furthermore, the influences of the ring size and the entangling modes on the topological entropy force are also investigated by examining the corresponding force-extension curves. This work, together with our previous work, might pave the path toward the complete formulation of static topological constraints.

## 1. Introduction

Polymer entanglement presents a big challenge for polymer physics [1]. Previously, de Gennes [2] and Edwards and Doi [3,4,5] developed the reptation theory or tube model to avoid directly describing the dynamic topological constraints involved in polymer entanglements and they had achieved great success in explaining experimental results in polymer rheology. Nevertheless, there are also weaknesses of the model, and many modified models based on the tube model, such as the contour-length fluctuations (CLF) model [6], the thermal constraint release (TCR) model, and the constraint release rouse (CRR) model [7,8,9,10,11], have been developed. Nevertheless, these modified models have not explicitly considered dynamic topological constraints in theory either, which greatly limits their wider applications [1]. Therefore, the development of a theoretical framework that can explicitly incorporate the dynamic topological constraints still remains an unresolved problem in polymer physics, which, unfortunately, is extremely difficult.

On the contrary, the problem of static topological constraints is less difficult, but thoroughly solving this problem might point out a way to eventually solve the dynamic-topological-constraint problem. Using an analogue with the Schrödinger equation, Edwards [12,13] solved the problem of a single chain entangled with a static and simple topological constraint—a pole. Even though the proposed approach is beautiful, unfortunately, it cannot be extended to cases involving multiple static topological constraints [14,15,16]. Our previous work [17] proposed a superspace approach to solve the static entanglement problem, where all possible entangling modes are mapped into a superspace with the inner structure characterized by a free group. This theoretical framework can, in principle, describe a polymer’s entanglement behavior with multiple static topological constraints. However, the calculations in our previous work only dealt with the case of multiple simple objects with genus zero (a polymer chain entangled with two poles in a 2D plane) and they did not consider complex-shaped topological constraints with a genus more than zero.

Therefore, this work is devoted to extending our previous work [17] to approach the static entanglement of a polymer entangled with a relatively more complex object with genus one—a ring-shaped object. We chose the ring-shaped object as the model topological constraint for three reasons. Firstly, mathematically, it should be the simplest object that has a genus of more than zero. Secondly, recently there have been more and more works, including experiments [18,19] and simulations [20,21,22], devoted to the investigation of the rheological properties of linear-ring polymer (or DNA) blends [18,20] or tadpole polymer melts [19] and they found that the ring-shaped structure would increase the viscosity of the polymer melts. A theoretical work about a polymer chain and a ring will help to understand the viscosity-increase phenomenon. Third, there are also many other examples in living systems and experiments that can be modelled as a polymer chain interacting with a ring-shaped object to some extent. For example, the interaction between a ring-shaped protein [23,24,25] and a polymer chain can be modelled as a polymer chain interacting with a ring-shaped object; while the interaction between a poly(ethylene glycol) (PEG) chain with cyclodextrins is another example [26,27,28]. Lastly, a mixture of a traditional polymer suspension with magnetic colloidal polymers [29,30,31] can be understood as the third example, where rings and more complex closed structures tend to form in order to minimize the magnetic flux.

This work is organized as follows. In the next section, the superspace approach based on the free group will be introduced first using a polymer chain entangled with multiple ring-shaped objects as an example. Then we will focus on the case of a polymer chain entangled with a ring-shaped object in an axisymmetric coordinate and present an alternating-direction implicit (ADI) scheme specially designed for the axisymmetric coordinate. In the result section, we will first study the entropic attractive force created by the inner freedom of the superspace due to the topological constraint of the ring-shaped object. The force-extension curves of pulling two ends of a polymer chain entangled with this ring-shaped object with a given entangling mode will be also studied.

## 2. Methods

### 2.1. Superspace Approach for Static Topological Constraints

To be more generic, we consider an ideal chain entangled with n ring-shaped objects and assume that there is no special interaction between the rings and the chain. These *n* ring-shaped objects are modelled as a toroid with the radius of the overall shape, *R*, and with the section area $b^{2}$ where b is the Kuhn length of the ideal chain. The partition function of this ideal chain entangled with these ring-shaped objects can be expressed as [12,29,30],

(1) $$Z=\int e^{-\int_{0}^{1}\frac{3Nb^{2}}{2}{\left|\frac{\partial R\left(s\right)}{\partial s}\right|}^{2}ds-\overset{n}{\underset{i=1}{\sum }}\int_{0}^{1}\int_{\Gamma_{i}}^{}\delta \left(R\left(s\right)-r\right)drds}DR\left(s\right),$$

where N is the chain length and $\int_{\Gamma_{i}}^{}\cdot dr$ integrates over the space occupied by the $i^{th}$ ring. This partition function is normally evaluated in terms of the end-integrated distribution function or propagator, q(r,s), according to Z=∫q(r,s=1)dr. Here, the propagator, q(r,s), is defined as

(2) $$\;q\left(r,s\right)=\int e^{-\int_{0}^{1}\frac{3Nb^{2}}{2}{\left|\frac{\partial R\left(\tau \right)}{\partial \tau }\right|}^{2}d\tau -\overset{n}{\underset{i=1}{\sum }}\int_{0}^{1}\int_{\Gamma_{i}}^{}\delta \left(R\left(\tau \right)-r^{\prime }\right)dr^{\prime }d\tau }\delta \left(R\left(s\right)-r\right)DR\left(s\right),$$

which can be obtained as the solution of the diffusion equation.

Note that the second term in the exponent of Equation (1) is used to incorporate the excluded-volume effect of the rings. However, as pointed out by our previous work [17], this term cannot fully describe the topological constraints of rings because even when the volume of $\Gamma_{i}$ approaches zero (with infinitely small section area) and the excluded-volume effect thus disappears, the topological constraint still exists due to the presence of these extremely ‘slim’ rings.

Therefore, our previous work [17] proposed the superspace approach to formulate the static topological constraints. The basic idea of the superspace approach is to expand the original space into a set of subspaces with each subspace corresponding to an entangling mode between the chain and the static constraints and each entangling mode corresponding to a group element of an *n*-generator free group. Here, *n* usually corresponds to the number of static constraints.

**Figure 1:**
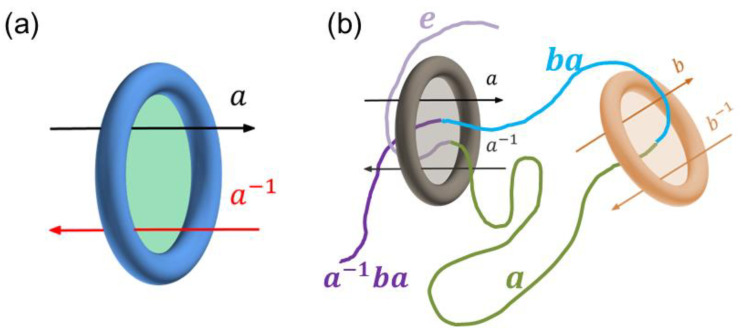
(**a**) In the superspace approach of n ring-shaped obstacles, the subspaces can be connected with a ‘portal gate’, which is actually the circular plane (green) of the ring. Crossing the gate from left to right, in this example, corresponds to a generator a of the n -generator free group, which is also a group element of this group. (**b**) A typical example of the entangling mode corresponding to the group element $a^{-1}ba$. Note that the light-purple chain segment is in the identity subspace e, the green chain segment is in the subspace a, the light-blue is in ba, and the purple is in $a^{-1}ba$.

A detailed definition of this superspace is referred to in ref. [17]. Here, we present an example of a chain entangled with two rings to demonstrate the relationship between the topological constraints and the *n*-generator free group. When the rings are presented, the space suddenly becomes different for the polymer chain. Each ring’s circular plane (green patch in Figure 1a) can be seen as a ‘portal gate’ connecting two different subspaces of the superspace. If the chain segment is now in the subspace g with g an element of the group, then it will enter the subspace ag as it crosses the gate from left to right and it will enter $a^{-1}g$ from right to left. For the two-ring case (Figure 1b), there will be two portal gates corresponding to the two generators of the free group 〈a, b〉. In this special example, the light-purple chain segment is in the subspace $g_{1}=e$ with e the identity element of the group, another end segment (purple) is in the subspace $g_{2}=a^{-1}ba$, and, therefore, the entangling mode of this chain conformation with respect to the rings is $g=g_{2}g_{1}^{-1}=a^{-1}ba$. Note that these two generators do not commute (e.g., $a^{-1}ba\neq b$). For simplicity, this work only considers a one-toroid case, and the free group of the superspace of the one-toroid topological constraint is isomorphic to the group of integers or isomorphic to the infinite cyclic group with group elements $\left\{\ldots a^{-2},\;a^{-1},e,a,a^{1},a^{2},\ldots \right\}.$

Accordingly, the original propagator q(r,s) will be expanded to an infinite number of propagators denoted by $q_{g}\left(r,s\right)$ with g a group element of the n-generator free group corresponding to an entangling mode. When r is not closed to the portal gate, the function $q_{g}\left(r,s\right)$ only propagates within the subspace g according to the diffusion equation,

(3) $$\frac{\partial q_{g}\left(r,s\right)}{\partial s}=\frac{Nb^{2}}{6}\nabla^{2}q_{g}\left(r,s\right),$$

Near the portal gate denoted by some generator a, the function $q_{g}\left(r,s\right)$ will propagate into the subspace ag or $a^{-1}g$, which actually determines the boundary conditions of Equation (3) (see Equations (8) and (9) in Section 2.2).

### 2.2. ADI Scheme in Cylindrical Coordinates and Boundary Conditions

As mentioned above, this work only considers a one-toroid case for simplicity, and in this case, Equation (3) can be numerically solved in a cylindrical coordinate parametrized by (r, z, θ), by assuming axial symmetry, which can be further reduced to 2D represented by (r, z). To be consistent with the axisymmetric coordinate, the ends of the chain must be placed on the axis z. Note that for a particle-based simulation of this system, it is inappropriate to employ this axisymmetric coordinate. Luckily, for probability-based theoretical calculations, the axisymmetric assumption is reasonable, and it will render the calculations easier.

In the calculations, we adopted the following alternating-direction implicit (ADI) scheme [31] to solve the diffusion equation,

(4) $$\left(1-\frac{\Delta s}{2}L_{R}\right)q_{g}^{*}\left(r,z,s\right)=\left(1+\frac{\Delta s}{2}L_{Z}\right)q_{g}\left(r,z,s\right)$$

(5) $$\left(1-\frac{\Delta s}{2}L_{Z}\right)q_{g}\left(r,z,s+\Delta s\right)=\left(1+\frac{\Delta s}{2}L_{R}\right)q_{g}^{*}\left(r,z,s\right),$$

where the discrete operators $L_{R}$ and $L_{Z}$ are defined in the following two equations,

(6) $$L_{Z}q_{g}\left(r,z,s\right)=\kappa \left(q_{g}\left(r,z-\Delta z,s\right)+q_{g}\left(r,s,z+\Delta z\right)-2q_{g}\left(r,z,s\right)\right)$$

(7) $$L_{R}q_{g}\left(r,z,s\right)=\{\begin{array}{cc} 4\kappa \left(q_{g}\left(\Delta r,z,s\right)-q_{g}\left(0,z,s\right)\right) & \left(r=0\right)\\ \kappa \left[\frac{1}{r}q_{g}\left(r-\Delta r,z,s\right)\left(q_{g}-\frac{\Delta r}{2}\right)+\frac{1}{r}q\left(q_{g}+\Delta r,z,s\right)\left(r+\frac{\Delta r}{2}\right)-2q_{g}\left(r,z,s\right)\right] & \left(r\neq 0\right) \end{array}$$

respectively, with Δr and Δz being the discrete steps along r and z, which are both set to the Kuhn length b in the calculations. Note that the operator $L_{R}$ defined in Equation (7) can ensure calculations will not encounter a numerical problem near r=0.

Moreover, the boundary conditions (BC) for the ‘portal gate’ can be added by properly modifying Equation (6). If the chain segment’s probability propagates from left to right through the gate, then Equation (6) is modified to

(8) $$L_{Z}q_{g}\left(r,z_{p},s\right)=\kappa \left(q_{g}\left(r,z_{p}-\Delta z,s\right)+q_{ag}\left(r,s,z_{p}+\Delta z\right)-2q_{g}\left(r,z_{p},s\right)\right)$$

where $z=z_{p}+\Delta z/2$ is the location of the portal gate and r in the equation should be smaller than the radius of the gate. Note that the second term on the right-hand side is the propagator in another subspace ag indicating that the probability can diffuse from subspace g to subspace ag through the gate at $z=z_{p}+\Delta z/2$. Accordingly, Equation (6) is modified to, for the probability propagating from right to left through the gate,

(9) $$L_{Z}q_{g}\left(r,z_{p}+\Delta z,s\right)=\kappa \left(q_{a^{-1}g}\left(r,z_{p},s\right)+q_{g}\left(r,s,z_{p}+2\Delta z\right)-2q_{g}\left(r,z_{p}+\Delta z,s\right)\right)$$

For the space occupied by the ring, the probability cannot diffuse inside this part of space, and on the surface of the ring, the reflective BC is adopted, as follows,

(10) $$L_{Z}q_{g}\left(r_{S},z_{S},s\right)=\kappa \left(2q_{g}\left(r_{S},z_{S}-\Delta z\right)-2q_{g}\left(r_{S},z_{S},s\right)\right)$$

where $\left(r_{S}+\Delta r/2,z_{S}+\Delta z/2\right)$ is assumed to be on the surface of the ring and $\left(r_{S},z_{S}+\Delta z\right)$ is assumed to be inside the ring. Equation (7) should be modified accordingly under the reflective BC.

## 3. Results

### 3.1. Attraction of the Ring to the Gaussian Chain

We first explore the statistical behavior of an ideal chain when dragging one of its two ends away from the ring. In the numerical calculations, one chain end will be fixed at z=0 along the central axis (r=0), which can be achieved by setting the initial condition to $q_{g}\left(r,z,s=0\right)=\delta \left(r\right)\delta \left(z\right)\delta_{g,e}$ for Equation (3).

**Figure 2:**
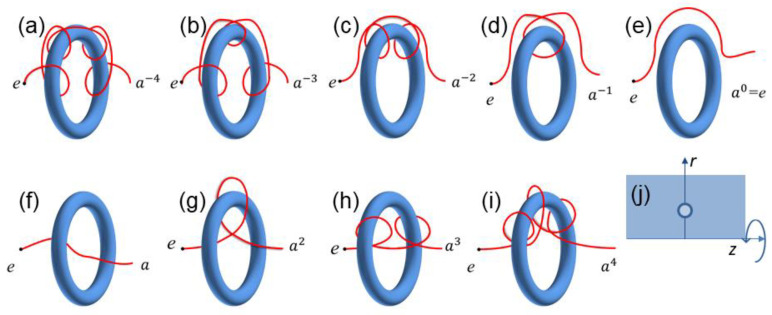
In evaluating the probability of the free end of an ideal chain being found in the subspace g, denoted by $P_{g}$, we explore nine possible subspaces or nine entangling modes (**a**–**i**). Note that another end of the chain is fixed at point in the subspace e with e the identity element of the infinite cyclic group. (**j**) Illustration of the (r, z) coordinate employed in this work. The blue circle represents the cross-section of the ring. The axis z is the central axis of the ring.

**Figure 3:**
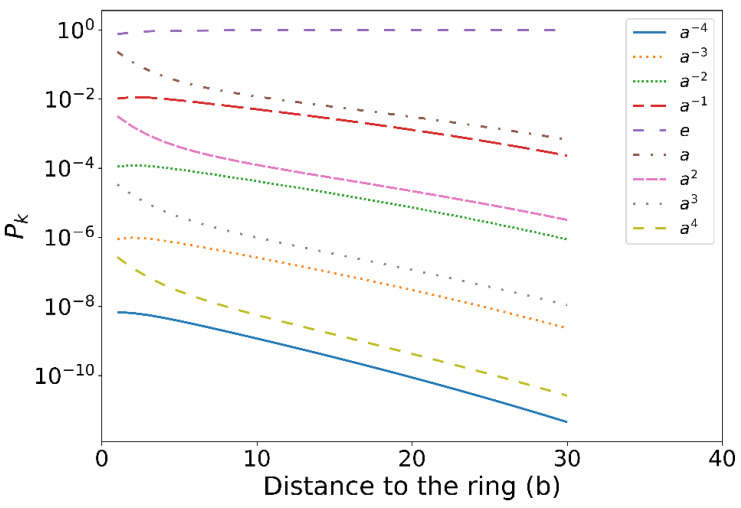
The probability of an ideal chain with the free end being found within the subspace $g=a^{k}$ and another end fixed at a point in subspace e. The x -axis shows the distance of the free end to the ring plane in the unit of Kuhn length b. Note that the probability function is normalized over g, i.e., $\underset{g}{\sum }P_{g}\left(d\right)=1$. Here, the ring with overall radius 3b and the fixed end of the chain are both fixed at (r=0 and z=0).

First, we compute the probability of the other free end of the chain found in the subspace g, denoted by $P_{g}$. We also investigate its dependence on the distance of the chain end to the ring (note that the probability is normalized over g, i.e., $\underset{g}{\sum }P_{g}\left(d\right)=1$). Figure 2a–i show the typical chain conformations entangled with the ring with the free end being found in nine different subspaces that have been explored in this work. Figure 3 shows that the probability $P_{g}\left(d\right)$ is extremely small ($<10^{-6}$) for $a^{k}$ with |k|≥4, which indicates, in the numerical calculations, that it is sufficient to only consider these nine subspaces rather than all the subspaces of the superspace. It is also interesting to find that for two entangling modes with the same winding number (brown dashed and orange dotted curves in Figure 3), the chain that winds the ring inward (see Figure 2i for an example) would have a smaller probability of finding the other end than the chain that winds the ring outward (see Figure 2b for an example). The reason for this phenomenon is likely because crossing the ring’s hole will decrease the probability, and even for entangling modes with the same winding number, the inward winding mode (e.g., Figure 2i) will cross the ring’s hole one more time compared to that of the outward winding mode (e.g., Figure 2b). It is more obvious when the winding number is zero that for the entangling mode of Figure 2f (outward winding), the chain has to cross the ring to reach the other side of the ring through a small ring’s hole, while for the chain in Figure 2e (inward winding), it can reach the other side much more easily.

**Figure 4:**
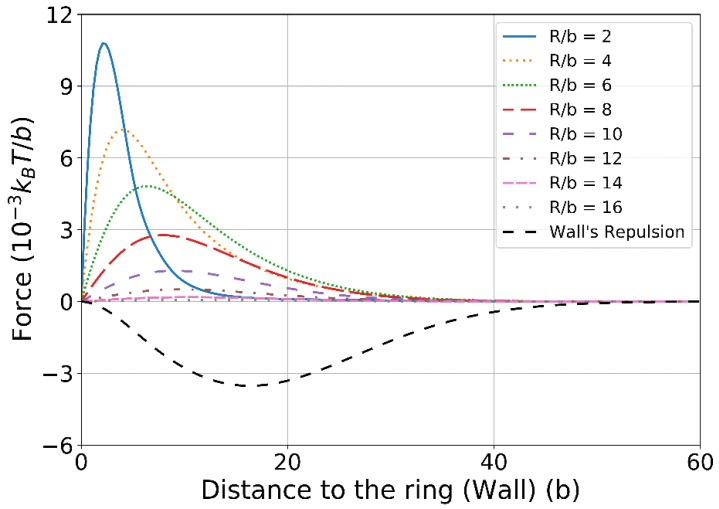
The entropic force felt by the chain when dragging one end of the chain away from the ring (solid lines) or the wall (dash line). Here, we set the force pointing in direction towards the ring or the wall as positive. In the legend, *R* denotes the ring‘s overall radius.

Second, we compute the entropic force exerted on the chain because of the presence of the ring, and the force is evaluated by $F\left(L\right)=\partial k_{B}T\ln Z\left(L\right)/\partial L$ with L being the distance of the fixed chain end to the ring and Z(L) being the partition function of the chain. When the chain is placed near a solid wall, the chain will be pushed away by the wall and the chain will feel a repulsive force (see the dashed curve in Figure 4) because the wall will reduce the conformational entropy of the chain and the chain will tend to leave the wall to increase the entropy. On the contrary, when the chain is near a ring, the chain will quickly find that there is an infinite number of subspaces created by the topological constraint of the ring and the chain segments can diffuse, through the portal gate, into these subspaces, which will, in turn, increase the conformational entropy of the chain. Therefore, the chain will be attracted to the ring due to the topological-entropy force (see Figure 4). Figure 4 also investigates the dependence of the topological-entropy force on the ring size and it is found that the peak of the force curve rises as the ring size decreases. This result is in accordance with our intuition that once the chain has been trapped by the ring with a small hole, it will be difficult to drag it out. This result also has an important implication for dynamical polymer entanglement in that the dynamical topological constraint will also attract the chain or will also become sticky to the chain because of the topological-entropy force discovered in this work. For example, it has been reported in several simulation works [19,21] that the viscosity of the linear-ring polymer blends will be significantly increased compared with the linear polymer melts. Experiments [19] also showed that tadpole polymer melts have a larger viscosity than those of linear polymers or pure-ring polymers because, as indicated by Figure 4 in this work, the tail of the tadpole will be attracted by the head of another tadpole, which will certainly increase the viscosity of the melt.

### 3.2. Force-Extension Curves of an Ideal Chain Entangled with a Ring

**Figure 5:**
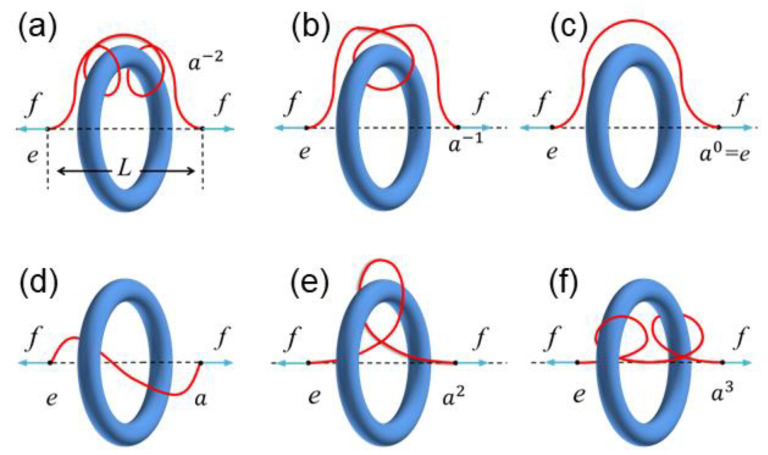
Typical chain conformations of six entangling modes that have been explored in this work. (**a**–**e**) show the entangling modes of $a^{-2}$, $a^{-1}$, e, a, $a^{2}$ and $a^{3}$, respectively; and in each entangling mode, the two ends of the chain with distance separation L are pulled by an external force f.

We also compute the force-extension curves of an ideal chain entangled with a ring at z=0 with six different entangling modes (Figure 5). In evaluating the force, we fix two chain ends at z=−L/2 and z=L/2, respectively, and compute its partition function Z(L); then the force can be computed as $F\left(L\right)=\partial k_{B}T\ln Z\left(L\right)/\partial L$. According to our previous work [17], $F\left(L\right)\sim \frac{3k_{B}Tl\left(L,R,R_{C},g\right)}{Nb^{2}}+\frac{Qk_{B}T}{l\left(L,R,R_{C},g\right)}$ with $l\left(L,R,R_{C},g\right)$ being the effective extension of the polymer chain, Q being the topological charge of the entangling mode, R being the overall radius of the toroid or the ring, and $R_{C}$ being the radius of the cross-section of the ring.

**Figure 6:**
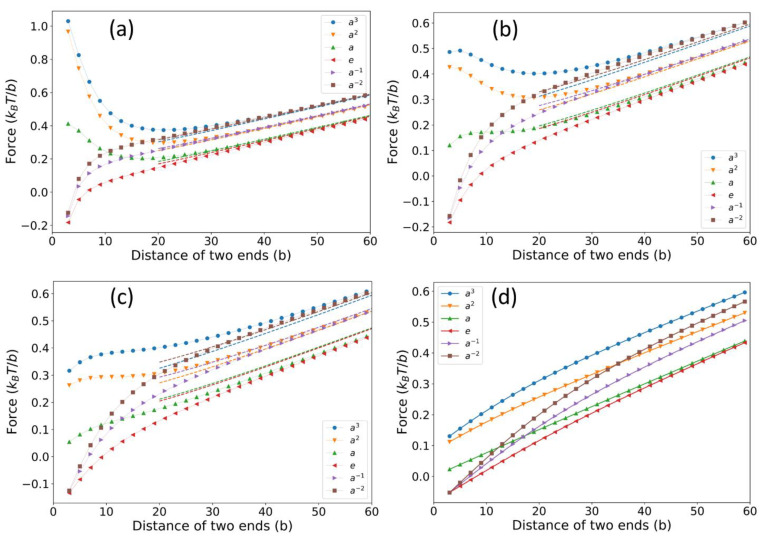
Force-extension curves of an ideal chain entangled with a ring with the overall radius of (**a**) R=2b, (**b**) R=4b, (**c**) R=6b, and (**d**) R=16b. Six different entangling modes (see Figure 5), corresponding to six curves in each plot, have been explored. The results obtained by superspace approach are given by full circles (with solid lines) while the results obtained by the blob model are given by dashed lines.

**Figure 7:**
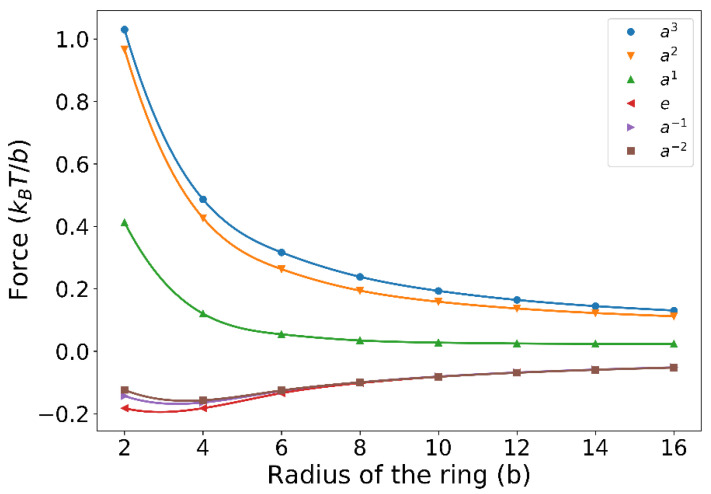
The influence of the overall radius of the ring on the entropic force of an ideal chain entangled with a static ring-shaped object with six different entangling modes. In this plot, the distance, L, between the two chain ends is 3b.

There are several prominent features of the force-extension curves shown in Figure 6 and Figure 7.

**Figure 8:**
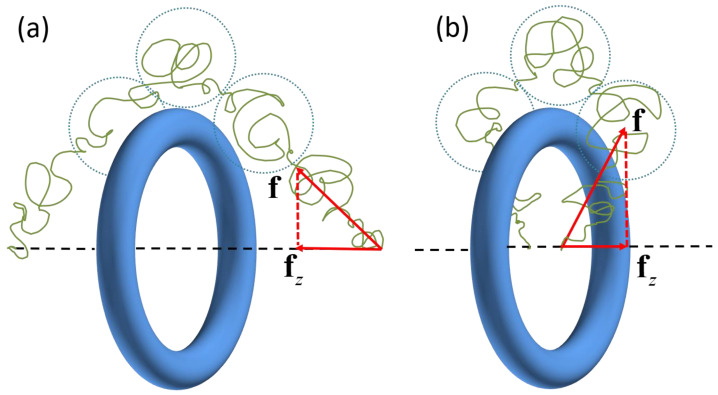
Schematic illustration of the force-direction reverse when L decreases for the outward-winding entangling modes with (**a**) for the large L region and (**b**) the small L region.

(i) The force-extension curves for large L are basically linear and they approximately satisfy the relation $F\left(L\right)\sim 3k_{B}Tl\left(L,R,R_{C},g\right)/Nb^{2}$ with $l\left(L,R,R_{C},g\right)$ being the effective extension of the polymer chain. Obviously, $l\left(L,R,R_{C},g\right)$ also depends on the entangling mode g or winding number of the chain on the ring. For example, winding the ring two times outward ($a^{-2}$) or inward ($a^{3}$) will both increase the l by approximately $4\pi R_{C}$ and it will accordingly increase the force by approximately $12k_{B}T\pi R_{C}/Nb^{2}$, which is actually the force gap between modes $a^{3}$ and e (see the blue curves and red curves in Figure 6). In fact, this feature can be fully captured by the blob model (dash lines) when the overall radius of the ring is small (Figure 6a) for the large L region. However, for a relatively large ring radius, the blob model is inaccurate (Figure 6b,c).

(ii) The force-extension behavior of inward winding (Figure 5e,f) differs greatly from that of the outward winding (Figure 5a,b) in the small L region. When the chain winds the ring inward, the force is pointing towards the ring and will be dramatically increased for a large winding number (see the blue curve in Figure 6a), which is similar to the force-extension curves of an ideal chain entangled with a pole [17]. In contrast, for outward winding, the force becomes repulsive (the red, purple, and brown curves in Figure 6a). This direction-reverse phenomenon of the entropic force is explained schematically in Figure 8. When it is outward winding and L is large (Figure 8a), the projection of the entropic recovery force on the axial is apparently pointing to the ring. On the contrary, when L is small, even though the recovery force is still pointing from the chain end, the direction of its projection on the axial has been reversed (Figure 8b). The linear behavior in the large extension region has been also witnessed by some other work [32], where they calculated the force-extension curves of a polymer chain with sliding links, which can be seen as ring-shaped objects. Meanwhile, for the small extension region, they only observed the force-decrease phenomenon similar to the curves in Figure 6d. The difference between our work and their results is mainly due to the fact that the models used in these two works are different and they have not considered the influence of complicated entangling modes.

(iii) By fitting the blob model or $F\left(L\right)\sim \frac{3k_{B}Tl}{Nb^{2}}+\frac{Qk_{B}T}{l}$ to the force-extension curves calculated by the superspace approach, the topological charge Q can be numerically determined, and they are Q(a)=0, Q(e)=0.5 and Q=1 for other entangling modes. It is interesting to find that the effective topological charge of the entangling mode does not depend on the winding number. This can be explained by the non-Gaussian perturbation argument in our previous work [17]. In our previous work, we showed that the number of the topological charge of a given entangling mode only depends on the number of independent non-Gaussian perturbation points, $N_{non-G}$, along the chain, and it can be proved that normally, $Q=N_{non-G}$. Here, the non-Gaussian perturbation is defined as follows: If one slightly drags or perturbs a chain segment away by Δl, which causes the effective extension to increase NOT by $c\Delta l^{2}/l$ with some coefficient c, then we call this perturbation non-Gaussian. In the ring-chain entanglement, there will be maximally one independent non-Gaussian perturbation point along the chain and, therefore, Q is, at most, 1.

(iv) We also compare forces of different entangling modes at small *L* when the ring size increases. It is found that when the ring size is small, force gaps between different entangling modes are relatively large and they become relatively small when the ring size is big (Figure 7).

## 4. Conclusions

In this work, the statistical mechanics of an ideal chain entangled with a static ring-shaped obstacle is studied by a superspace approach based on the free group theory. It is found that when the chain is near the ring, the freedom of the subspaces created by the topological constraint of the ring will attract the chain by the topological-entropy force and this theoretical result can explain the viscosity-increase phenomenon observed in recent simulations [20] and experiments [18,19]. The force-extension behavior of the ideal chain entangled with the ring has been also investigated and it is found that the force curves of the inward entangling modes behave quite differently from those of the outward entangling modes for the small extension region.

This work demonstrates that the superspace approach can be extended to study the polymer chain entangled with relatively complicated objects, such as rings of genus one. In our future works, we will consider a polymer chain entangled with multiple ring-shaped objects or with other complicated objects of a bigger genus.

## Data Availability

Not applicable.
